# Supplementary material for: Enhancement of Visible-Light Photocatalytic Degradation of Tetracycline by Co-Doped TiO2 Templated by Waste Tobacco Stem Silk
Source: Molecules. 2023 Jan 2;28(1):386. doi: 10.3390/molecules28010386 (PMC9822450; doi:10.3390/molecules28010386)
Supplement: Supplementary file 1 [file molecules-28-00386-s001.zip › molecules-2103508-supplementary.pdf]

Supplementary Materials

Enhancement of visible-light photocatalytic degradation of tetracycline by Co-doped TiO_2 templated by waste tobacco stem silk

Quanhui Li [1], Liang Jiang [1,*], Yuan Li [1], Xiangrong Wang [2], Lixia Zhao [1], Pizhen Huang [1], Daomei Chen [1], Jiaqiang Wang [1,*]

1. School of Materials and Energy, Yunnan Province Engineering Research Center of Photocatalytic Treatment of Industrial Wastewater, School of Chemical Sciences & Technology, School of Engineering, National
2. Kunming Academy of Eco-Environmental Sciences, Kunming 650032, China

* Correspondence: jiangliang@ynu.edu.cn (L.J.); jqwang@ynu.edu.cn (J.W.)

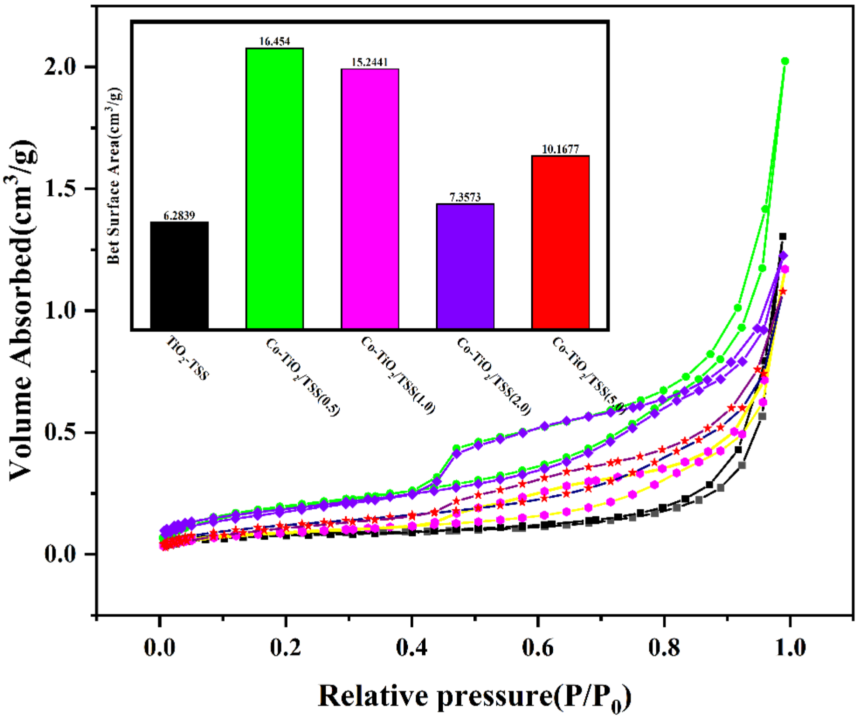

Figure S1. N_2 adsorption-desorption isotherm and the BET specific surface areas (inset) of as-prepared samples.

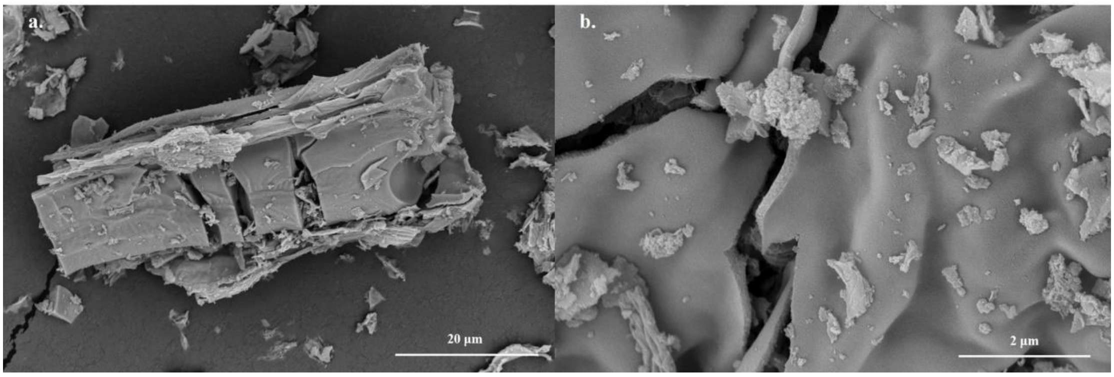

Figure S2. SEM images of Co-TiO_2/TSS(5.0)

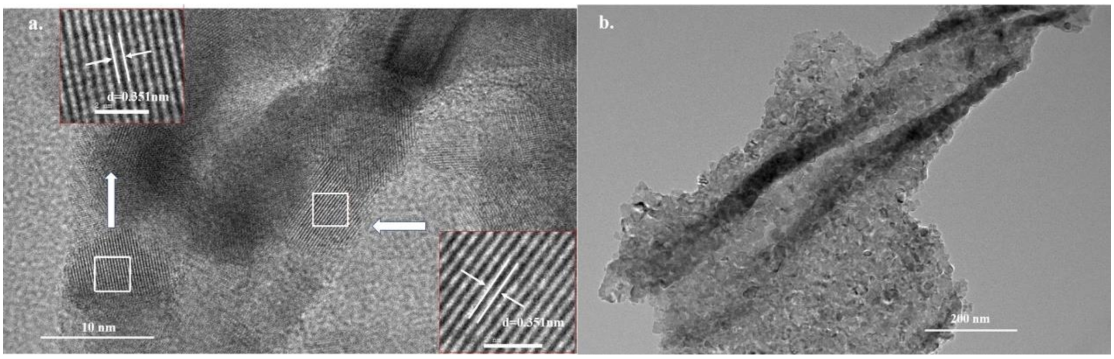

Figure S3. TEM images of Co-TiO_2/TSS(5.0)

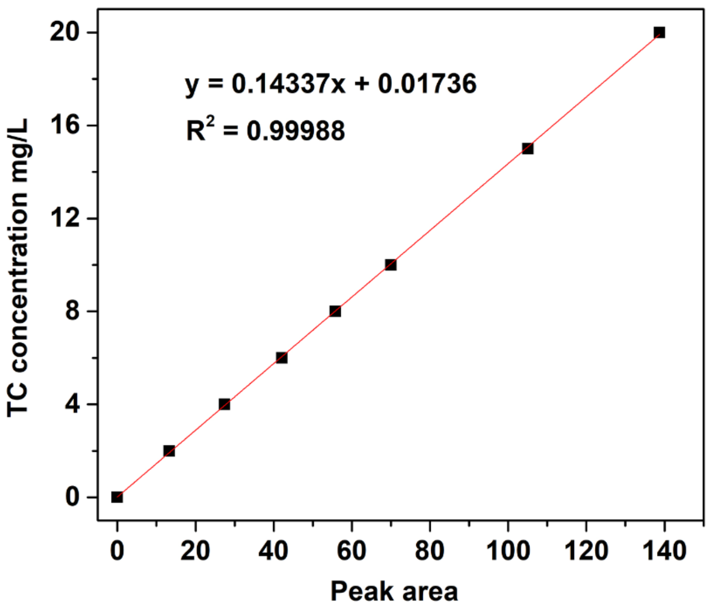

Figure S4. Linear relationship between peak area and TC concentration.

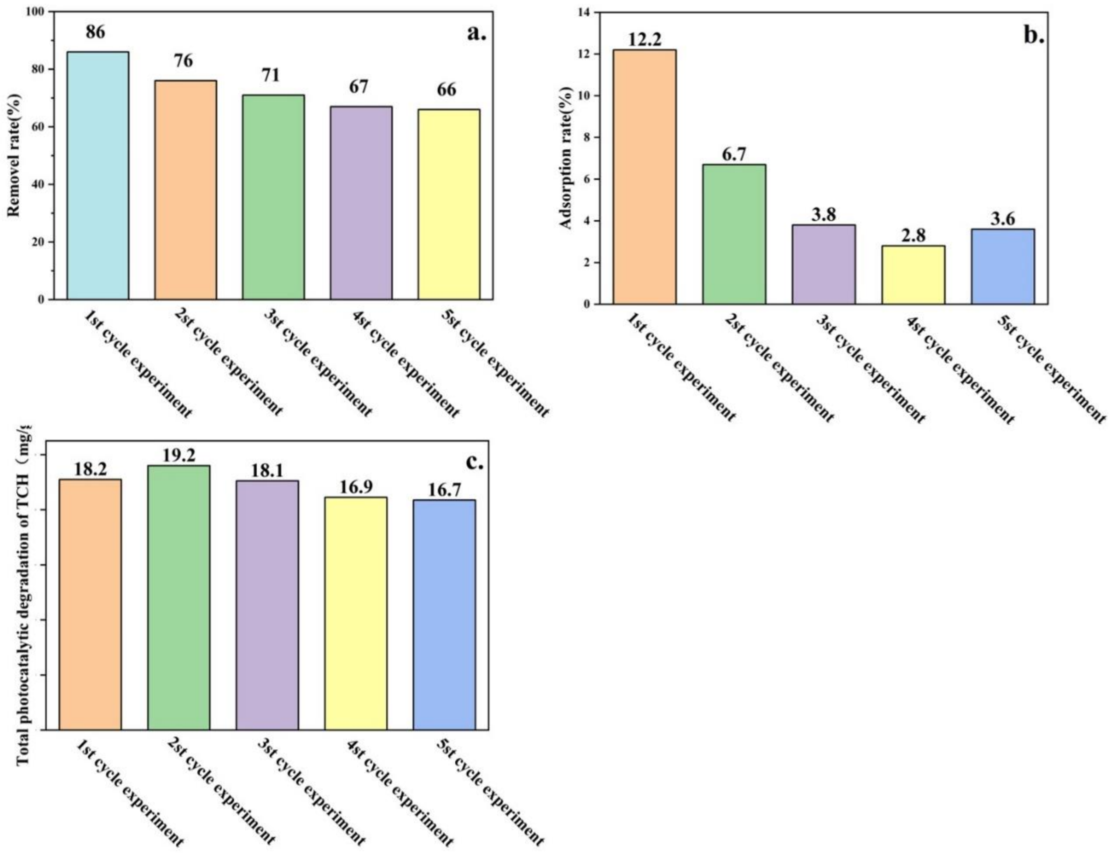

Figure S5. (a) Removal rate of TCH, (b) adsorption rate of TCH, and (c) rate of total TCH removal over Co-TiO_2/TSS under visible light during the cycling experiments.
